# Influencing public acceptance of artificial intelligence (AI) in healthcare delivery

**DOI:** 10.3389/fdgth.2025.1664345

**Published:** 2026-01-13

**Authors:** Selin Aras, Calvin Drakos, Vineesha Manimangalam, Moiz Ali Nasir, Christina Burns, Davey Smith, Ozlem Equils

**Affiliations:** 1 MiOra, Los Angeles, CA, United States; 2 Department of Medicine, University of California, San Diego, La Jolla, CA, United States

**Keywords:** artificial intelligence, behavioral models, health anxiety, healthcare delivery, public trust, social cognitive theory, technology adoption, theory of planned behavior

## Abstract

**Introduction:**

Despite the potential of artificial intelligence (AI) to transform healthcare delivery and reduce costs, adoption remains uneven across populations. Understanding the demographic, behavioral, and cognitive factors influencing public willingness to use AI-powered health tools is critical for equitable implementation. This study examined determinants of AI adoption in healthcare among adults in the United States (U.S.).

**Methods:**

A cross-sectional survey was conducted between March and June 2024 using convenience sampling across the U.S. The study included 568 adult respondents recruited via Qualtrics. The survey assessed demographic characteristics, digital health behaviors, self-reported health status, cognitive and attitudinal factors, and behavioral intentions related to AI use in healthcare. Logistic regression models were used to examine associations between predictors and willingness to adopt AI, with z-tests for subgroup comparisons and Bonferroni correction applied for multiple hypothesis testing.

**Results:**

The sample was predominantly female (66.7%) and Hispanic/Latino (50.7%), with moderate income and education levels. Older age was negatively associated with AI adoption (*β* = −0.029), males were less likely to use AI than females (*β* = −0.388), and income was positively correlated with AI adoption (*β* = 0.096). Trust in AI was substantially lower than trust in physicians: 14.6% trusted ChatGPT's diagnosis for serious illness compared with 92.3% trusting physicians, and 17.1% versus 96.4% for specialist referrals. Telehealth use strongly predicted AI adoption (*β* = 1.012), while lower self-rated mental health was associated with higher AI use (*β* = −0.254). Uninsured participants reported higher trust in AI diagnostic capabilities than insured participants (57% vs. 43%, *p* < 0.05). Ethnic differences were observed, with Asian participants reporting higher AI usage rates than Hispanic participants (16.49% vs. 5.56%, *p* < 0.05).

**Discussion:**

AI adoption in healthcare is shaped by the interaction of demographic, socioeconomic, and cultural factors. While AI has the potential to expand healthcare access, adoption patterns reflect existing disparities in healthcare access and trust. Trust emerged as a central determinant, with AI functioning as a compensatory tool when traditional healthcare access is limited. Given the U.S.-specific context, findings should be interpreted as exploratory and may not generalize to other healthcare systems. These results highlight the need for future research on transparency, digital literacy, and structural barriers to support equitable implementation of healthcare AI.

## Introduction

Over the past decade, healthcare technologies have advanced at an unprecedented pace. Among these developments, AI has emerged as a transformative force in healthcare delivery, with the potential to significantly improve diagnostic accuracy, optimize treatment planning, and enhance administrative efficiency (1). Projections indicate that by 2026, AI applications could reduce annual U.S. healthcare costs by approximately $150 billion (2). Supplementing this estimate, a study assessing the broader adoption of AI found that $360 billion could be saved annually through AI's potential for medical error reduction (3). Ranging from machine learning algorithms to natural language processing and predictive analytics, AI-powered tools are becoming increasingly integrated into clinical workflows, promising to improve outcomes for both physicians and patients (4).

**Table 1:** Demographic profile of survey (*N* = 568).

Category	Response	Percentage
Household Income	$0–$22,000	21.70%
	$22,000–$89,450	43.70%
	$89,451–$190,750	24.10%
	$190,751–$384,200	6.00%
	>$384,200	4.60%
Education Level	Less than High School	3.70%
	High School Diploma	13.00%
	Some College	22.90%
	2-year Degree	11.60%
	4-year Degree	32.00%
	Masters	11.10%
	Doctorate	3.30%
	Technical or Trade School	2.30%
Ethnicity	Hispanic/Latino	50.70%
	Asian	17.10%
	White/Caucasian	11.60%
	Black/African American	10.20%
	2+ Ethnicities	5.10%
	Other*	5.30%
Sex	Female	66.70%
	Male	33.30%
Political Affiliation	Undecided	35.20%
	Liberal/Left Wing	32.20%
	Moderate	22.90%
	Conservative/Right Wing	9.70%
Healthcare-Related Career	Work in healthcare/science	32.50%
	Do not work in healthcare/science	67.50%
Health Insurance	Has health insurance	87.30%
	Does not have health insurance	12.70%
Age	Median age	31 years
	Age range	18–83 years
Trust in Healthcare System	Completely trust	8.80%
	Trust most of the time	43.60%
	Neutral/ambivalent	34.00%
	Don't trust that much	11.50%
	Don't trust at all	2.10%
Doctor Visit Frequency	For a yearly check-up:	50.0%
	To follow-up on something I have been seen for before	35.4%
	When I am sick or have new symptoms	58.9%
	Never	8.5%
AI Usage	Use AI for at least one purpose	45.10%
	Do not use AI	54.90%
AI Use Cases	School	27.40%
	Work	22.10%
	Socialization	14.80%
	Health/personal care	9.20%

*Other includes Middle Eastern, Native Hawaiian or Other Pacific Islander, South Asian, American Indian or Alaska Native.

**Table 2:** Logistic regression coefficients for predicting community AI Use for healthcare.

Predictor	Coefficient (*β*)	CI 2.5%	CI 97.5%	SE	*p*-value	OR
Age	−0.029	−0.0740	−0.0090	0.0163	0.006	**0** **.** **971**
Gender identity (encoded)	−0.388	−1.0920	0.0000	0.3396	0.042	**0** **.** **678**
Mental health rating	−0.254	−0.5857	0.0000	0.1510	0.026	**0** **.** **776**
Income (encoded)	0.096	−0.1498	0.3206	0.1157	0.392	**1** **.** **101**
Education (encoded)	−0.0035	−0.1652	0.1569	0.0848	0.902	**0** **.** **996**
Use of mental health apps	−0.349	−1.2785	0.2432	0.4051	0.158	**0** **.** **705**
Use of exercise apps	0.000	0.0000	0.0000	0.0000	0.000	**1** **.** **000**
Use of menstrual tracking apps	0.260	−0.3230	0.8129	0.2777	0.284	**1** **.** **297**
Use of electronic chart system	0.000	0.0000	0.0000	0.0000	0.000	**1** **.** **000**
Use of biosensors	0.000	0.0000	0.0000	0.0000	0.000	**1** **.** **000**
Use of telehealth/telemedicine	1.012	0.4347	1.6379	0.3093	0.000	**2** **.** **751**
Not having a private place to use telemedicine	−0.2560	−1.4065	0.2682	0.4144	0.154	**0** **.** **774**
Increased level of trust in the healthcare system and government organization health recommendations	−0.1936	−0.5177	0.1163	0.1707	0.200	**0** **.** **824**

CI, confidence interval; SE, standard error; OR, odds-ratio.

Bold values indicate statistically significant associations (*p* < 0.05).

**Table 3:** Community use and attitudes towards AI.

Category	Count (*N*)	Proportion (%)
Uses AI (any purpose)	262	46.2%
Trusting a Physician's Diagnosis for a Serious Illness	524	92.3%
Trusting ChatGPT's Diagnosis for a Serious Illness	83	14.6%
Trusting a Physician's Referral for a Specialist	548	96.4%
Trusting ChatGPT's Referral for a Specialist	97	17.1%

AI, artificial intelligence.

Despite this transformative promise, the adoption of AI in healthcare remains uneven across populations. A general acceptance of AI is influenced by an interplay of cognitive, behavioral, and structural factors, including digital literacy, perceptions of usefulness, provider trust, and concerns surrounding algorithmic fairness and transparency (5). Surveys consistently show that while Americans welcome AI for administrative or supportive tasks, they are more hesitant about its role in clinical decision-making (6). Additionally, systematic review by Beets et al. (7). found that although many U.S. adults acknowledge AI's potential, but concerns about data privacy, transparency, and trust persist. Similarly, Witkowski et al. (8) reported that one-third of survey respondents trusted AI to provide diagnoses, citing a fear of losing the “human touch” in healthcare; however, similar to Tyson et al.'s (6) findings, most respondents were comfortable with AI's administrative duties, such as scheduling appointments.

Moreover, recent national data adds to the complexity of U.S. adults' attitudes toward AI in healthcare. A 2025 nationally representative survey found that only about 19.55% of respondents believed AI would improve their personal doctor-patient relationship or healthcare affordability. Notably, individuals with higher levels of trust in providers displayed more positive expectations of AI as a strong tool for healthcare practices (9). Similarly, an earlier survey of over 900 U.S. adults reported that openness to AI varied significantly by demographic factors such as age and education, and that personality traits like trust and psychosocial factors strongly influenced whether individuals viewed AI in healthcare as beneficial or concerning (10). These findings underscore that public perceptions of AI are not only shaped by technological promise but also by relational and systemic concerns that draw from one's demographic and psychological determinants.

Hesitations of AI also transcend to a systematical level. Organizational studies show uneven deployment of AI across U.S. health systems. For example, Poon et al. (11) noted that while generative AI tools such as ambient documentation have gained traction, many clinical AI applications face barriers due to regulatory uncertainty, immature tools, and financial concerns. This variation highlights that adoption is also influenced by regulations and institutional readiness.

## Limitations of existing literature

While several studies (6, 7, 10) provide valuable insights, they also carry limitations needing further studies to resolve. Many of these studies (8, 10) relied on regional or convenience samples, limiting generalizability. Others (7, 9) used inconsistent survey methods, which complicates cross-comparisons. Large-scale surveys, such as Tyson et al. (6), risk framing effects that may amplify public fears of AI, while cross-sectional designs, including those of Nong and Ji (9) and Antes et al. (10), limit the ability to determine whether one factor directly causes another. Recent scoping reviews add to this evidence: Botha et al. (12) mapped the perceived benefits of AI, identifying improvements in diagnostic sensitivity, workflow efficiency, and error reduction, while also showing that evidence is scattered and often disease specific. In contrast, Botha et al. (13) mapped the perceived threats, including unpredictable errors, loss of the “human touch,” insufficient regulatory frameworks, algorithmic bias, and data privacy concerns. Together, these reviews reinforce that much of the existing literature remains fragmented, addressing isolated issues such as trust, usability, or fairness without integrating how these factors interact with demographic and psychosocial determinants. To address this gap, we applied an integrated framework from the behavioral sciences. Key perspectives from Social Cognitive Theory, which studies the role of personal experience, environmental context, and observational learning in shaping behavior (14), the Theory of Reasoned Action (TRA) and the Theory of Planned Behavior (TPB) which emphasize that behavioral intentions are driven by attitudes, perceived social norms, and perceived behavioral control (15), and trust-centered models of technology acceptance suggest that trust plays a critical mediating role, particularly in contexts where users must rely on automated or opaque systems (16). In addition, the Health Belief Model and the Short Health Anxiety Inventory framework were incorporated to capture the influence of perceived health risks, anxiety, and individual health status on decision-making and behavior (17). This allows us to create a unified model of AI adoption. Unlike prior studies that examine single determinants in isolation, such as trust, demographics, or perceived usability, our approach synthesizes cognitive, demographic, psychosocial, and trust-related predictors within a single analytic framework. This integrated perspective makes it possible to examine how these factors interact rather than treating them as separate or unrelated contributors. By focusing on the general United States adult population and including individuals who actively use generative AI for health information, who represent an understudied and increasingly relevant subgroup, this study extends prior work that has relied heavily on regional, convenience, or profession-specific samples. Together, these design choices differentiate our study from existing literature even though a cross-sectional survey format was necessary.

By situating this study within these theoretical frameworks, we aim to provide a more comprehensive understanding of how demographic, cognitive, and trust-related factors collectively shape public willingness to adopt AI-driven healthcare tools in the U.S. population.

## Methods

### Study design

This study employed a cross-sectional survey design, a method that collects data once per participant to assess associations between variables within a defined population (18). The design was used to examine factors associated with the willingness of adults in the U.S. to adopt AI in healthcare. While cross-sectional surveys are common in this field, our study builds on prior work by applying a conceptually integrated measurement framework that captures demographic, cognitive, psychosocial, and trust-related variables simultaneously, allowing for a more comprehensive assessment of AI adoption than has been achieved in earlier single-factor studies. The study protocol and the survey instrument were reviewed and approved as exempt by the Western-Institutional Review Board (Protocol #1755408).

### Sampling and data collection

Participants were recruited between March and June 2024 through MiOra health education networks (19) and Qualtrics research panels. Eligibility criteria included being 18 years or older and residing in the U.S.; the final sample ranged in age from 18 to 83 years. A total of 689 responses were collected; after excluding incomplete or invalid submissions, 568 complete entries were retained for analysis. Recruitment through Qualtrics ensured a broad national reach, while MiOra's health education channels facilitated participation from individuals with prior exposure to digital health. All participants provided informed electronic consent prior to participation. Surveys were conducted online, collected anonymously, and no personal identifiers were retained.

### Measures

Survey items (Supplementary Appendix 1) were developed to assess demographic characteristics, digital health behaviors, self-reported health status, cognitive and attitudinal factors toward AI, and behavioral intentions.

Instrument development was informed by prior literature on technology acceptance and trust in healthcare AI (7, 8, 10), which provided conceptual grounding for items measuring perceived usefulness, reliability, and trust. A similar behavioral framework, the Technology Acceptance Model (TAM), was also applied in a recent study by Svestkova A et al. (20), which examined community acceptance of generative AI for health purposes. Although the investigators built their hypothesis and model on prior research about medical professionals' trust in medical AI, their data were drawn from community participants. Importantly, their findings confirmed that trust was a central mediator of willingness to use AI. Our work extends this framework by focusing specifically on non-medical populations using ChatGPT for health information, where the determinants of trust may differ substantially.

Items were further refined through (a) feedback from representative community members affiliated with MiOra health education programs, who reviewed the survey for clarity, readability, and cultural relevance; and (b) input from academic experts in behavioral sciences and digital health, who evaluated content validity. The survey was not piloted as a standalone study; however, iterative refinement through this process ensured face and content validity prior to launch. This iterative process helped establish face validity by ensuring that survey items were clear and culturally appropriate for respondents, and content validity by confirming that the items adequately covered the intended domains, e.g., demographic, cognitive, and attitudinal factors. Feedback from community members focused on clarity and cultural relevance, while expert reviewers emphasized conceptual alignment and comprehensiveness.

Demographic measures included age, gender identity, racial and ethnic background, formal educational attainment, and annual household income (reported according to an aggregated version of the 2024 tax bracket) (21). Digital health behavior questions assessed prior experience with AI-supported applications, including telehealth platforms, wearable biosensors, mental health apps, and menstrual tracking tools. Health-related variables included self-reported physical health, measured by “How would you rate your physical health?”; mental health, measured by “How would you rate your mental health?”; and frequency of interactions with the healthcare system.

To measure cognitive variables, the survey included items assessing attitudes toward AI (e.g., perceived usefulness, reliability), perceived social norms (e.g., whether healthcare providers or peers support AI), and perceived behavioral control (e.g., confidence in using AI-based tools). Trust in AI was measured using a comparative item assessing whether participants trusted AI or human providers more when making health-related decisions. A willingness to use AI was operationalized through items capturing openness to AI-generated recommendations and comfort with AI involvement in care. Representative items included “If AI/ChatGPT told you to see a specialist, would you take it seriously?” to assess attitudes toward AI and “Do you agree with the statement ‘A doctor is less likely to understand my healthcare needs than AI/ChatGPT?’ to assess perceived social norms.”

In addition to these domains, survey items were intentionally aligned with constructs from Social Cognitive Theory (SCT), the Theory of Reasoned Action (TRA), the Theory of Planned Behavior (TPB), trust-centered technology acceptance frameworks, and the Health Belief Model (HBM). SCT concepts such as behavioral capability and environmental constraints were reflected in items assessing prior use of digital health tools (e.g., telehealth, mental health apps, biosensors) and access to privacy for telemedicine. TRA/TPB constructs were operationalized through items capturing attitudes (“If AI/ChatGPT told you to see a specialist, would you take it seriously?”), subjective norms (“A doctor is less likely to understand my healthcare needs than AI/ChatGPT”), and perceived behavioral control (comfort understanding medical information sheets, familiarity with digital tools). Trust-centered acceptance models were addressed through comparative trust items contrasting physician vs. AI diagnoses and referrals. HBM-related constructs were incorporated through items measuring perceived susceptibility and severity (self-rated physical and mental health) and perceived barriers (difficulty understanding health information sheets, lack of privacy for telemedicine, and level of trust in the healthcare system). These theoretical mappings allowed us to integrate demographic, cognitive, psychosocial, and trust-related determinants of AI adoption within a unified behavioral framework.

Because ChatGPT was used as the reference point for all AI-related survey items, participants may have evaluated a familiar, general-purpose chatbot rather than a clinically validated medical AI system. ChatGPT was selected because it is currently the most widely used large language model, but its consumer-facing nature may have shaped respondents' perceptions of both AI use and AI trust in a healthcare context.

### Statistical analysis

All analyses were conducted using Python software. Descriptive statistics, including means, standard deviations, and frequencies, were used to summarize demographic characteristics, health behaviors, and cognitive and attitudinal measures. Logistic regression models were used to examine associations between demographic (age, assigned sex, gender identity, income), health-related (mental health rating), and behavioral predictors (digital health behaviors) and the dependent variable, willingness to use AI for health-related purposes, operationalized as responses to “Yes, I use AI/ChatGPT for health and/or personal care” (22). Multi-response survey items were converted into separate binary variables to indicate each selected option. Categorical predictors were encoded numerically or dummy coded as appropriate (23). Predictors with zero variance (constant values across all respondents) were excluded prior to fitting the model to ensure stable coefficient estimation (24). All entries with missing data for the variables used in the analysis were removed (25). Chi-square tests and two-sample *z*-tests were used to compare categorical variables across subgroups, and a Bonferroni correction was applied to adjust for multiple comparisons (23, 26, 27). Logistic regression model convergence was ensured using maximum iterations set to 1,000, and statistical significance was defined at *α* = 0.05 (28, 29).

The dependent variable for all logistic regression models was the response to the item “Yes, I use AI/ChatGPT for health and/or personal care,” coded as 1 = yes and 0 = no. Predictors were created directly from survey items: age was treated as a continuous variable; self-rated mental health and trust in the healthcare system were coded as ordinal variables; and each option in multi-select digital health behavior items (e.g., “I use telehealth/telemedicine,” “I use a menstrual cycle tracking app,” “I don't have a private place to use telemedicine”) was converted into its own binary indicator (0 = not selected, 1 = selected). All categorical demographic variables, including gender identity, income, and race/ethnicity, were dummy-coded prior to analysis (23). Logistic regression models produced log-odds coefficients (*β*); for interpretability, we also exponentiated these values to obtain odds ratios (OR = *e^β^*), which are reported alongside the *β* coefficients in the results table (30).

## Results

### Sample characteristics

The sample included 568 respondents (Table 1). The median age was 31 years (IQR: 23–41, range: 18–83). Over half identified as Hispanic/Latino (50.7%), followed by Asian (17.1%), White/Caucasian (11.6%), and Black/African American (10.2%); 5.1% identified as two or more ethnicities, and 5.3% as Middle Eastern, Native Hawaiian/Pacific Islander, South Asian, or American Indian/Alaska Native. Women comprised 66.7% of the sample.

Educational attainment was varied: 3.7% had less than high school, 13.0% held a high school diploma, 22.9% some college, 11.6% a 2-year degree, 32.0% a 4-year degree, and 16.7% had graduate or professional training. Household income ranged from $0 to over $384,200, with 43.7% in the $22,000–$89,450 bracket and 21.7% earning below $22,000.

Politically, 35.2% identified as undecided, 32.2% as liberal, 22.9% as moderate, and 9.7% as conservative. About one-third (32.5%) worked in healthcare or science, and 87.3% had health insurance.

Healthcare engagement varied: 58.9% sought care when experiencing new symptoms, 50.0% attended yearly check-ups, and 35.4% attended follow-ups; 8.5% reported never visiting a doctor. Trust in the healthcare system was moderate overall, with 43.6% reporting trust “most of the time,” 34.0% neutral, 8.8% high trust, and 13.6% low trust.

Nearly half (45.1%) used AI for at least one purpose. Among AI users, common uses included school (27.4%), work (22.1%), and socialization (14.8%). Only 9.2% reported using AI specifically for health or personal care.

### Demographic factors and AI adoption

Logistic regression identified several demographic predictors of AI use for health purposes (Table 2). Younger age was significantly associated with higher adoption (*β* = –0.029, *p* = 0.006), and men were less likely than women to use AI for health information (*β* = –0.388, *p* = 0.042). Income showed a small positive coefficient, but this association did not reach statistical significance (*β* = 0.096, *p* = 0.39), indicating that income was not a reliable predictor of AI use in this sample. Consistent with descriptive trends, individuals with lower educational attainment (high school or below) were less likely to use AI (60% vs. 47%, *p* = 0.0188) and also reported more difficulty understanding printed health information (12.9% vs. 29.9%, *p* < 0.001).

AI use varied across racial and ethnic groups: Asian (16.5%), African American (15.5%), and South Asian (16.7%) respondents reported the highest adoption rates, whereas White (7.8%) and Hispanic/Latino (5.9%) respondents reported the lowest. The only statistically significant comparison was between Asian and Hispanic participants (*p* < 0.001). Despite nearly half the sample using AI for some purpose, health-related use remained low (9.2%), suggesting that general familiarity with AI does not automatically translate to medical adoption.

### Trust in AI as an intermediary factor

Trust strongly differentiated attitudes toward AI in healthcare (Table 3). 92.3% trusted a physician's diagnosis for a serious illness compared to 14.6% who trusted ChatGPT, and 96.4% trusted physician referrals compared to 17.1% who trusted ChatGPT recommendations. This indicates a clear preference for human clinical judgment.

Digital familiarity influenced adoption: telehealth users were significantly more likely to use AI for health (*β* = 1.012, *p* < 0.001), and menstrual tracking app use showed a positive but nonsignificant association (*β* = 0.260, *p* = 0.284). Lack of a private space for telemedicine was associated with lower AI use, though not significantly (*β* = –0.256, *p* = 0.154). Increased level of trust in the healthcare system and government organization health recommendations was negatively associated with AI use for health purposes but did not reach a level of significance, meaning that those with decreased level of trust had a higher correlation of use with AI for health purposes (*β* = −0.194, *p* = 0.200), consistent with patterns of seeking information outside traditional healthcare. Several features—use of exercise apps, electronic chart systems, and biosensors—had coefficients of 0. These zero coefficients arose because the corresponding predictors had no variance after preprocessing in the dataset, meaning that none of the respondents who used AI for health purposes reported using these features, rendering them uninformative for the model. Consequently, these predictors do not have any statistical significance. This was expected to be a statistical artifact.

### Health status and anxiety as moderators

Lower self-rated mental health was associated with significantly greater AI use for health purposes (*β* = –0.254, *p* = 0.026), suggesting that individuals experiencing psychological strain may turn to AI as an accessible supplementary resource (Table 2). Conversely, individuals already using mental health apps were less likely to use AI for broader health purposes, though this pattern was nonsignificant (*β* = –0.349, *p* = 0.158). Across these variables, we observed that uninsured participants, those with lower self-rated mental health, those reporting difficulty understanding medical information, and those with lower trust in the healthcare system showed higher reliance on AI tools, indicating patterns consistent with compensatory use when traditional healthcare access is limited.

## Discussion

The findings from this study underscore the complex interplay of demographic, technological, and social factors influencing AI adoption in healthcare. Younger age, higher income, distrust in the current medical system, and prior use of digital health tools such as telemedicine and menstrual tracking apps were positively associated with adoption, while barriers such as lack of private space, lower education levels, and limited trust in AI tools constrained engagement. Although AI tools hold significant promise for expanding access and improving outcomes, current adoption patterns reveal that pre-existing differences—particularly those linked to socioeconomic status, education, and race—are mirrored in the digital health landscape. These disparities highlight both the potential of AI to contribute to improvements in healthcare delivery and the importance of understanding inequities in digital access and trust. Because attitudes toward AI are shaped by insurance structure, cost barriers, and cultural norms unique to the U.S. healthcare system, these results should be interpreted as context-bound rather than globally generalizable.

### Socioeconomic barriers to AI adoption

Although the direction of the coefficient suggested that higher income may be associated with greater AI use, this relationship was not statistically significant. This contrasts with previous research that showed that financial insecurity may reduce opportunities for engaging with innovative health solutions (31). Similarly, population-level analyses during the COVID-19 pandemic found that lower-income groups reported reduced uptake of digital health technologies compared to higher-income counterparts (32), Several factors may explain this discrepancy, including differences in our outcome variable (AI use for health specifically vs. general digital health engagement), the demographic composition of our sample, and the presence of stronger predictors (e.g., telehealth use, trust, mental health) that may attenuate the independent effect of income.

From the perspective of the Theory of Planned Behavior, these socioeconomic patterns suggest that individuals with higher income and education may experience greater perceived behavioral control, making them more confident in navigating AI tools (15). Social Cognitive Theory further supports this interpretation by emphasizing the role of environmental resources and prior experience, both of which are more available to individuals with higher socioeconomic status (14). From an HBM perspective, limitations such as difficulty understanding medical information sheets function as perceived barriers, which may reduce engagement with traditional care and increase reliance on AI as an alternative source of health information (17).

### Educational attainment and AI literacy

Our results also indicated that participants with lower levels of education were less likely to use AI tools and reported more difficulty understanding printed health information materials. This finding is consistent with previous studies that have shown education to be a strong predictor of digital health engagement. For instance, other studies have demonstrated that higher educational attainment is associated with greater digital health literacy and more frequent use of health technologies (33). Taken together, these results highlight that disparities in educational access may translate directly into disparities in the ability to effectively adopt and benefit from AI-driven healthcare platforms. This suggests that digital health platforms must simplify user interfaces and language to accommodate varying levels of health and digital literacy (34). The ability to comprehend post-visit information, already a barrier to traditional healthcare, appears to be further amplified in the context of AI (35). This aligns with Social Cognitive Theory, which posits that behavioral capability and self-efficacy shape technology engagement (14). Individuals with lower health and digital literacy may perceive fewer skills or resources to use AI effectively, which directly limits perceived behavioral control as described in the Theory of Planned Behavior (15).

### Cultural influences on AI trust and use

In our quantitative analysis, we found that Hispanic participants were significantly less likely than Asian participants to adopt AI-based health tools indicating potential cultural or contextual influences shaping engagement with AI. This aligns closely with recent qualitative evidence. A study by Kraft et al. (36) conducted focus groups with Hispanic and Latinx adults, revealing concerns around mHealth tools, which frequently include AI features such as unfamiliarity with technology, privacy apprehensions, and fears of overreliance on automated systems. Participants emphasized the value of human oversight, expressing discomfort with entirely automated interventions and concern that AI tools might not adequately account for cultural norms or nuanced personal contexts.

Together, these findings suggest that lower rates of AI adoption among Hispanic respondents may be partially driven by cultural and community-level apprehensions. The qualitative insights underscore that perceived trustworthiness, need for personal connection, and cultural appropriateness are important mediators of AI acceptance, factors that are not easily captured through quantitative measures alone. These findings illustrate how subjective norms, a central construct in the Theory of Planned Behavior, may shape AI use across cultural groups (14). Communities with stronger norms emphasizing personal interaction with healthcare providers may perceive AI as inconsistent with those expectations. Social Cognitive Theory similarly suggests that community-level models, norms, and shared experiences influence individual behavioral intentions (15).

Gender differences also emerged in our analysis. Men were significantly less likely than women to use AI tools for healthcare, a result consistent with longstanding evidence that men are generally less likely to engage in proactive health-seeking behaviors compared to women (37). This suggests that AI adoption patterns may not only reflect cultural differences across ethnic groups but also broader socialized norms around gender and healthcare engagement. The Theory of Reasoned Action provides a useful lens here: if men hold weaker subjective norms around proactive help-seeking, they may be less inclined to adopt AI tools designed to support health behaviors. This reflects broader attitudinal and normative influences rather than purely technological ones (15).

### Trust as a mediator in AI adoption

Trust in AI emerged as a key mediating factor in healthcare AI adoption, with individuals who reported lower trust in AI being less likely to use it for health purposes. This finding aligns with the Technology Adoption Model, which posits that external factors, including environmental infrastructure shape technology uptake (38).

A recent scoping review further supports this, identifying trust as a significant catalyst for AI adoption in healthcare (39) Increasing transparency and explainability in AI decision-making, alongside close collaboration with trusted healthcare providers, will be key to building public confidence in these tools (39). Our results that trust predicts AI adoption align with recent evidence emphasizing human-centered and participatory approaches in healthcare AI, where embedding trust and equity into design is critical for improving adoption (8, 40).

Additionally, decreased trust in the healthcare system showed a higher correlation with AI use for health purposes, though this relationship did not reach statistical significance. This finding aligns with previous research demonstrating that people with lower trust in official healthcare sources are more likely to use and trust alternative information sources such as blogs and social media (41). While that study predated widespread AI adoption, it suggests a consistent pattern of seeking secondary information sources when trust in traditional healthcare is diminished.

This is consistent with trust-centered technology acceptance models, which emphasize that trust is a prerequisite for forming positive attitudes toward automated systems (16). In the Theory of Planned Behavior, trust operates through attitudes and perceived control, shaping behavioral intention (15). Social Cognitive Theory also emphasizes observational learning and prior experience as foundations of trust, which explains why individuals familiar with digital health tools showed higher adoption (14).

### Influence of mental health on AI adoption in healthcare

In our findings, lower self-rated mental health was associated with a significantly higher likelihood of turning to AI tools for health purposes. This pattern suggests that individuals experiencing elevated psychological distress may be more inclined to use AI as a supplemental or accessible resource, especially when traditional care appears less accessible. A parallel German study similarly found that psychological distress moderated the relationship between perceived usefulness of AI and actual mental health app usage, suggesting that distress itself can drive greater engagement with digital tools (42). This pattern aligns with the Health Belief Model, which predicts that individuals with heightened perceived vulnerability or health-related concern may be more motivated to seek supplemental health information (17). It also reflects cognitive appraisal processes described in health anxiety frameworks, where distress can increase engagement with accessible information sources such as AI (17).

Conversely, prior use of mental health apps showed a negative, but non-significant, association with the use of broader AI health tools. This may reflect a phenomenon where individuals already using established digital mental health solutions feel a degree of satisfaction or trust in those services, reducing their motivation to explore AI alternatives. A related study in a Portuguese university cohort found that mental health app use was significantly associated with perceived stress and ongoing mental health concerns, indicating that known app usage may substitute for broader AI tool exploration (43).

### AI as a compensatory tool for limited healthcare access

In addition to these individual predictors, the patterns observed in this study suggest that AI may function as a compensatory healthcare resource for individuals who face barriers to traditional care. Participants who were uninsured, who reported lower trust in the healthcare system, or who had difficulty understanding printed medical materials demonstrated greater reliance on AI tools. Similarly, higher use among individuals with lower self-rated mental health indicates that psychological barriers may also prompt greater engagement with AI when in-person care feels less accessible or less supportive. These trends collectively imply that AI may be used as an alternative source of healthcare guidance when structural, informational, or psychological constraints limit engagement with conventional healthcare options.

### Interpretation of AI tools by participants

An important consideration in interpreting these findings is how participants understood the AI referenced in the survey. Because ChatGPT was used consistently across items, ranging from personal health information seeking to hypothetical diagnostic or referral scenarios, respondents may have evaluated AI through the lens of a familiar, general-purpose chatbot rather than a medically validated clinical tool. ChatGPT's popularity made it a practical anchor for assessing public attitudes, yet its consumer-facing design differs substantially from supervised diagnostic or decision-support systems used in healthcare. As a result, lower trust in ChatGPT for clinical tasks may reflect skepticism toward consumer AI rather than toward regulated medical AI. This distinction suggests that our trust estimates may underestimate how the public would respond to clinically validated AI tools integrated into provider workflows.

### Future directions and considerations

Findings from this study surface several themes that warrant deeper examination in future work. Patterns related to digital literacy, transparency, and structural barriers suggest directions for continued inquiry into how people evaluate and engage with emerging healthcare technologies. These exploratory observations can help inform future research that examines how such factors might shape policy discussions around the development and implementation of AI tools. Further research across diverse settings may clarify how different groups interact with these technologies and what influences their trust and willingness to use them.

### Limitations and future research

While this study offers valuable insights, several limitations must be acknowledged. First, the use of Qualtrics as a survey platform resulted in convenient sampling that may limit the generalizability of the findings. Hispanic or Latino respondents comprised more than half of the sample, creating the possibility of overrepresentation bias. This skew may have influenced subgroup comparisons, for example, observed differences in Hispanic participants' AI adoption could be partially confounded by their high proportion in the dataset and their socioeconomic characteristics. Future work could employ weighting procedures or stratified sampling to balance demographic representation. Although the sample size of 568 participants was adequate for logistic regression analyses, it remains small relative to the broader U.S. population it aims to represent. This, combined with the use of convenience sampling and the demographic skew of the sample, limits the extent to which these findings can be generalized. The size and composition of the sample also do not capture the full heterogeneity of the U.S. healthcare landscape, further constraining generalizability.

Second, reliance on self-reported data introduces the possibility of response bias and social desirability bias, particularly for questions about technology use, trust, and health behaviors. Respondents may have underreported barriers or overstated positive attitudes toward AI tools to align with perceived norms.

Third, the cross-sectional design precludes any claims of causality. While associations between demographic, behavioral, and attitudinal variables and AI adoption were identified, longitudinal studies are needed to assess how these relationships evolve over time and to establish temporal directionality.

Fourth, the choice of ChatGPT as the reference point for AI use may have shaped participant responses. Given its widespread media coverage and public visibility, ChatGPT may not be perceived as interchangeable with other AI health tools, introducing potential framing effects that could bias adoption rates or trust.

Fifth, although our study shares certain methodological constraints with prior research, such as the use of self-reported data and a cross-sectional design, we sought to address a major conceptual limitation noted in the current literature. Prior studies often examine single determinants of AI acceptance in isolation, which results in fragmented explanatory models. In contrast, our study integrates multiple behavioral science frameworks and examines demographic, cognitive, psychosocial, and trust-related variables within one unified model. This approach helps reduce conceptual fragmentation and provides a more comprehensive view of the factors shaping AI adoption in healthcare.

Finally, although the sample was diverse across several sociodemographic categories, it may not fully capture the heterogeneity of experiences across all subgroups. Future research should incorporate both longitudinal designs and qualitative methods to more deeply explore how demographic context, trust, and cultural factors shape the evolving landscape of AI use in healthcare. Additionally, because the study reflects attitudes shaped within the U.S. healthcare system, the findings may not generalize to countries with different structures, cultural norms, or access barriers.

## Conclusion

This study provides new insight into the demographic, behavioral, and cultural factors that shape public adoption of AI in healthcare. Key findings indicate that age, gender, income, and mental health status significantly influence adoption patterns, while prior engagement with digital health tools, such as telehealth and menstrual tracking apps, also contributes to openness toward AI-based healthcare. At the same time, barriers such as lack of privacy, lower education levels, and cultural differences underscore the persistence of structural inequities in the digital health landscape. Importantly, the analysis revealed that trust functions as a central mediator of AI use, amplifying or constraining adoption across diverse subgroups.

Taken together, these findings offer insights that can help generate hypotheses for future research on how demographic, structural, and attitudinal factors shape engagement with healthcare AI tools. The patterns observed here highlight areas for context-specific investigation, including how transparency, digital literacy, cultural factors, and trust influence public perceptions of AI. Understanding these dynamics more deeply may support future discussions about the development and implementation of AI systems in ways that reflect the needs and concerns of diverse U.S. communities. Despite using a cross-sectional approach similar to earlier studies, this work advances the field by integrating multiple behavioral science perspectives and examining how demographic, cognitive, and trust-related factors jointly influence public adoption of AI in healthcare.

Future research should move beyond cross-sectional analyses to employ longitudinal and experimental designs that clarify causal pathways between trust, demographic characteristics, and AI adoption. Additionally, qualitative studies could provide deeper insight into cultural and psychosocial factors underlying adoption behaviors, particularly among underrepresented groups. Expanding analyses to include a wider range of AI tools beyond ChatGPT would also improve generalizability and account for differences in public perceptions of various technologies. Together, these efforts will advance understanding of how to foster equitable, sustainable, and culturally sensitive integration of AI in healthcare.

## Data Availability

The raw data supporting the conclusions of this article will be made available by the authors, without undue reservation.
