# Correction: Influencing public acceptance of artificial intelligence (AI) in healthcare delivery

**DOI:** 10.3389/fdgth.2026.1895761

**Published:** 2026-07-02

**Authors:** Selin Aras, Calvin Drakos, Vineesha Manimangalam, Moiz Ali Nasir, Christina Burns, Davey Smith, Ozlem Equils

**Affiliations:** 1 MiOra, Los Angeles, CA, United States; 2 Department of Medicine, University of California, San Diego, La Jolla, CA, United States

**Keywords:** artificial intelligence, behavioral models, health anxiety, healthcare delivery, public trust, social cognitive theory, technology adoption, theory of planned behavior

The reference for 10 was erroneously written as Antes AL, Burrous S, Sisk BA, Schuelke MJ, Keune JD, DuBois JM. Exploring perceptions of healthcare technologies enabled by artificial intelligence: an online, scenario-based survey. BMC Med Inform Decis Mak. (2021) 21:221. doi: 10.1186/s12911-021-01573-5. It should be Antes AL, Burrous S, Sisk BA, Schuelke MJ, Keune JD, DuBois JM. Exploring perceptions of healthcare technologies enabled by artificial intelligence: an online, scenario-based survey. BMC Med Inform Decis Mak. (2021) 21:221. doi: 10.1186/s12911-021-01586-8.

The reference for 31 was erroneously written as Weissman JS, Stern R, Fielding SL, Epstein AM. Delayed access to health care: risk factors, reasons, and consequences. Am J Med. (1991) 91(2):114–22. doi:10.7326/0003-4819-114-4-325. It should be Weissman JS, Stern R, Fielding SL, Epstein AM. Delayed access to health care: risk factors, reasons, and consequences. Ann Intern Med. (1991) 114(4):325–331. doi:10.7326/0003-4819-114-4-325.

The reference for 38 was erroneously written as Taherdoost H, Mohamed N, Madanchian M. Navigating technology adoption/acceptance models. Procedia Comput Sci. (2024) 237:833–40. doi: 10.1016/j.procs.2024.05.091. It should be Taherdoost H, Mohamed N, Madanchian M. Navigating technology adoption/acceptance models. Procedia Comput Sci. (2024) 237:833–40. doi: 10.1016/j.procs.2024.05.172.

The original version of this article has been updated.

