# Supplementary material for: Influencing public acceptance of artificial intelligence (AI) in healthcare delivery
Source: Front Digit Health. 2026 Jan 13;7:1664345. doi: 10.3389/fdgth.2025.1664345 (PMC12853031; doi:10.3389/fdgth.2025.1664345)
Supplement: Supplementary file 1 [file Datasheet1.pdf]

Survey Questions

Demographic Information

1. **How old are you?**

2. **Which ethnicity do you most identify with?**
   ( ) White or Caucasian
   ( ) Asian
   ( ) Native Hawaiian or Other Pacific Islander
   ( ) Middle Eastern
   ( ) South Asian (such as India, Pakistan, Maldives)
   ( ) Black or African American
   ( ) American Indian or Alaska Native
   ( ) Hispanic or Latino
   ( ) Two or more ethnicities

3. **What is your assigned sex at birth?**
   ( ) Male
   ( ) Female
   ( ) Prefer not to say

4. **What is your gender identity?**
   ( ) Man
   ( ) Woman
   ( ) Non-binary
   ( ) Other

5. **What is your sexual orientation?**
   ( ) Heterosexual
   ( ) Gay
   ( ) Lesbian
   ( ) Bisexual
   ( ) Other

6. **What is your total household income?**
   ( ) $0 - $22K
   ( ) $22K - $89K
   ( ) $89K - $190K
   ( ) $190K - $364K
   ( ) $364K+

7. **What is your personal political affiliation?**
   ( ) Liberal
   ( ) Conservative
   ( ) Moderate
   ( ) Undecided

8. **What is your highest level of education completed?**
   ( ) Less than high school
   ( ) High school diploma or equivalent
   ( ) Some college
   ( ) Bachelor's degree
   ( ) Master's degree
   ( ) Higher degree

Career and Healthcare Access

9. **Is your career related to healthcare or science (physician, nurse, researcher, etc.)?**
   ( ) Yes
   ( ) No

10. **If you are employed, can you take paid time off to see a doctor?**
   ( ) I am not employed
   ( ) Yes
   ( ) No

11. **Do you have health insurance?**
   ( ) Yes
   ( ) No

Health and Wellness

12. **How would you rate your physical health?**
   ( ) Very good
   ( ) Good
   ( ) Average
   ( ) Poor
   ( ) Very poor

13. **How would you rate your mental health?**
    ( ) Very good
    ( ) Average
    ( ) Very poor

14. **When do you go to the doctor? (Select all that apply)**
    [ ] When I have new symptoms
    [ ] Follow-up on something I have been seen for before
    [ ] For yearly checkups
    [ ] Never

15. **Have you used an urgent care or the emergency room to get healthcare?**
    ( ) Yes, less than 5 times
    ( ) Yes, 5-10 times
    ( ) Yes, more than 20 times
    ( ) Never

16. **If you answered "Yes" to the previous question, please select all that apply:**
    [ ] I try to see my doctors before going to urgent care or ER
    [ ] I call my doctor/nurse before going to urgent care or ER
    [ ] I do not call my doctor/nurse before going to urgent care or ER
    [ ] I check the internet to decide whether I should go to urgent care

17. **Please rate your level of trust in the healthcare system and government organizations (CDC, FDA, etc.) health recommendations.**
    ( ) I do not trust them at all
    ( ) I don't trust them that much
    ( ) In the middle
    ( ) I trust them most of the time
    ( ) I trust them completely

Technology and Health Information

18. **Please select all that are true:**
    [ ] I use telehealth/telemedicine
    [ ] I don't have a private place to use telemedicine
    [ ] I use Amazon Health
    [ ] I use mental health apps
    [ ] I use exercise apps (Nike Training Club, Apple's Health/Fitness, etc.)
    [ ] I use a menstrual cycle tracking app
    [ ] I use the electronic chart system/portal of my provider (MyChart, etc.)

[ ] I use biosensors (smartwatch, smart ring, glucometer, etc.)

19. **Do you read the information sheets (after-visit summary, medication/procedure pamphlets, etc.) your doctor or pharmacist gives you?**
( ) I don't read it
( ) I read some of it
( ) I read most of it
( ) I read all of it
( ) Someone else reads it for me

20. **How useful do you find the information sheets?**
( ) They are useful for me most of the time
( ) They are useless and not relevant for me most of the time

21. **How comfortable are you in understanding the information sheets?**
( ) I don't understand
( ) I barely understand
( ) I understand half of it
( ) I understand most of it
( ) I understand all of it

22. **How do you feel about the format of the information sheets? (Select all that apply)**
[ ] I would not change the format
[ ] I wish the information came as a short video/Reel that I could watch
[ ] I wish the information came in another language
[ ] I wish the information was easier to understand
[ ] I wish the information was shorter/less words

23. **How would you most like to get information about taking care of your health? (Select all that apply)**
[ ] Reading words on a screen
[ ] Reading words on paper
[ ] Watching a video
[ ] Listening to audio only (podcast, radio, etc.)
[ ] Talking to my doctor or clinic staff

24. **The search engine I use the most is:**
( ) Google
( ) Bing
( ) Microsoft Edge
( ) Yahoo
( ) Other

25. **I look on the internet for health information ___. (Select all that apply)**
 [ ] To stay healthy
 [ ] To better manage my health issues/diseases
 [ ] To find information on an urgent health concern or illness
 [ ] To help family members or friends
 [ ] To find information on sensitive topics like STDs, reproductive health, pregnancy, or mental health
 [ ] Other (please specify)

26. **Have you ever cancelled or not made an appointment to see a healthcare professional for a health concern because you found information about it online?**
 ( ) Yes
 ( ) No

27. **If you answered "Yes" to the previous question, why? (Select all that apply)**
 [ ] I was able to address my concern
 [ ] It saved me time/money
 [ ] Other (please specify)

28. **Have you ever used artificial intelligence (AI) technology such as ChatGPT? (Select all that apply)**
 [ ] Yes, I use AI/ChatGPT to help with school
 [ ] Yes, I use AI/ChatGPT to help with work
 [ ] Yes, I use AI/ChatGPT for fun and socialization
 [ ] Yes, I use AI/ChatGPT for health and/or personal care
 [ ] Yes, I use AI/ChatGPT for other reasons (please specify)
 [ ] No
 [ ] No, I don't use it but would like to (please fill in the blank)

Attitudes Toward AI and Healthcare

29. **If your doctor told you to see a specialist, would you take it seriously?**
 ( ) Yes
 ( ) No

30. **If AI/ChatGPT told you to see a specialist, would you take it seriously?**
 ( ) Yes
 ( ) No

31. **If your doctor diagnosed you with a serious disease, would you trust that response?**
 ( ) Yes

( ) No

32. **If AI/ChatGPT diagnosed you with a serious disease, would you trust that response?**
    ( ) Yes
    ( ) No

33. **Do you agree with the statement "A doctor is less likely to understand my healthcare needs than AI/ChatGPT"?**
    ( ) Agree completely
    ( ) Somewhat agree
    ( ) Neutral/disagree
    ( ) Strongly disagree

34. **Do you agree with the statement "I feel more comfortable asking AI/ChatGPT questions on sensitive/embarrassing topics (vaccines, sexual health, etc.) than my doctor"?**
    ( ) Agree completely
    ( ) Somewhat agree
    ( ) Neutral/disagree
    ( ) Strongly disagree

35. **If AI/ChatGPT told you at the beginning of the chat that it cannot give you referrals for laboratory tests, X-rays/MRIs, or a prescription for medication, would you continue to use the AI/ChatGPT for health information?**
    ( ) Yes
    ( ) No

Follow-Up

36. **If you agree to be contacted for a follow-up survey, please leave your email below:**
    (Open text box)
